# The 100 top-cited meta-analyses of diagnostic accuracy in radiology journals: a bibliometric analysis

**DOI:** 10.1186/s13244-020-00936-w

**Published:** 2020-11-23

**Authors:** Kaspar L. Yaxley, Minh-Son To

**Affiliations:** 1 Centre for Evidence-Based Medicine, University of Oxford, Oxford, UK; 2 Division of Surgery and Perioperative Medicine, Flinders Medical Centre, Bedford Drive, Bedford Park, SA 5042 Australia; 3 College of Medicine and Public Health, Flinders University, Bedford Park, SA Australia

**Keywords:** Meta-analysis, Bibliometrics, Medical imaging

## Abstract

**Purpose:**

To identify the 100 top-cited meta-analyses of diagnostic accuracy studies published in radiology, medical imaging and nuclear medicine journals.

**Methods:**

A PubMed search with pre-defined criteria was performed. The 100 top-cited articles meta-analyses were retrieved, using a custom Python script and the Scopus Application Programming Interface (Elsevier). Publication, citation and affiliation details were extracted from each meta-analysis. No formal statistical analysis was performed.

**Results:**

The top meta-analysis was cited 394 times, the 100th meta-analysis 38 times. The USA was the top country represented in the papers (33 meta-analyses) followed by The Netherlands, China and Germany. The journal *Radiology* published 24 studies. The most common modality reported was positron emission tomography (PET) or PET computed tomography (36 instances), followed by magnetic resonance imaging (30 instances) and computed tomography (27 instances). Cardiac (19 meta-analyses), abdominal (18 meta-analyses), followed by neurological (12 meta-analyses) investigations were the most frequently encountered in the top 100 cited meta-analyses.

**Conclusions:**

The 100 top-cited meta-analyses encompass a broad range of imaging modalities and body regions. This may comprise a useful resource for identifying influential evidence-based diagnostic accuracy information in radiology.

## Introduction

Rapid advancements in medical imaging technologies have resulted in a corresponding proliferation of diagnostic modalities becoming available to clinicians for investigating many medical conditions. Choice of modality is often influenced by factors including cost, local availability, familiarity and patient characteristics (e.g. age and co-morbidities). However, diagnostic performance remains fundamental to appropriate selection as it provides information on the likelihood of true and false positives, likelihood of true and false negatives, and post-test probability of disease, all of which exhibit a strong relationship with downstream health outcomes. Choice of modality in a particular clinical situation is also often associated with a trade-off between these variables. For example, screening tests should be cost-effective, sensitive and non-invasive, but come at the expense of false positives. Understanding diagnostic accuracy is therefore critical for clinical decision making.

Diagnostic performance may be assessed by a number of parameters, including sensitivity and specificity, positive and negative predictive values, likelihood ratios, diagnostic odds ratios, and areas under the receiver operator characteristic curve [1]. These measures are especially relevant to diagnostic radiology, given the central role radiologists play in aiding clinicians in reducing diagnostic uncertainty by selecting the most appropriate imaging modalities and interpreting imaging findings. However, owing to the sheer number of diseases, imaging modalities, and the rapid expansion of the biomedical literature, keeping abreast of all such diagnostic information can be challenging for the practicing radiologist and other clinicians.

Meta-analyses of diagnostic test accuracy use statistical techniques for combining findings from multiple studies, providing pooled estimates of diagnostic accuracy measures such as sensitivity and specificity and an estimate of the uncertainty associated with these. These may provide more precise estimates of the diagnostic performance of imaging modalities compared to what might be obtainable from a single diagnostic accuracy study.

Although such studies provide synthesised, quantitative information on a certain topic, the onus still lies with the reader to identify the most relevant literature. Moreover, there are limited systematic reviews on meta-analyses of diagnostic accuracy in radiology and medical imaging, to our knowledge [2].

Bibliometric analyses offer one approach for identifying key studies and is a type of literature analysis comprising a collection of quantitative and statistical tools for evaluating the quality and impact of the literature associated with a certain topic or field [3]. It is a useful technique for revealing linkages among research articles and the utility of published work to researchers working in a particular field. Bibliometric analysis is founded on the assumption that the most important research findings are published in academic journals and that research is predominantly based on studies previously published in such journals [4]. Citation analysis is a commonly employed bibliometric method which attempts to quantify the impact of a study by the number of citations it has received. Such analysis may also permit an assessment of the impact of specific authors and their affiliated institutions, research journals and countries of origin. Citation counts are influenced by factors such as the level and hierarchy of evidence [5], the presence of statistically significant results [6, 7], and title length [8]. Indeed, similar findings have been corroborated in the radiology literature [9].

Although a bibliometric analysis does not provide a detailed review on a topic of interest, it may enable the reader to quickly and conveniently identify the influential articles in that topic. The purpose of our study was therefore to perform a bibliometric analysis and identify the 100 top-cited meta-analyses of diagnostic accuracy published in radiology, medical imaging and nuclear medicine journals.

## Materials and methods

The authors declare no conflict of interests. Research ethics approval was not required for this bibliometric study.

### Search strategy

The literature search was performed in PubMed. The search filter incorporated terms for retrieving meta-analyses and was limited to those journals listed in the category “Radiology, Nuclear Medicine and Imaging” by *Journal Citation Reports* [10]. The search was restricted to dates from the 1 January 2005 through 31 December 2019, since the landmark paper on bivariate analysis of sensitivity and specificity was published in 2005 [11]. Full details of the search string are provided in Additional file 1. The search was performed on the 1 April 2020.

### Study identification and data extraction

Both authors independently screened the title and abstract of studies identified from the search. Only meta-analyses of diagnostic accuracy studies in radiology were included in the study. For inclusion, studies had to perform a quantitative meta-analysis involving at least one imaging modality (e.g. x-ray, computed tomography, magnetic resonance imaging, ultrasound), be focused on a specific disease or condition, and report at least one diagnostic performance metric such as sensitivity, specificity, accuracy, odds ratio, likelihood ratio or area under the receiver operator characteristic curve. Studies identified by the original literature search but were excluded included reviews of diagnostic accuracy which did not perform a formal meta-analysis (e.g. due to inadequate data), studies concerned with prediction or prognosis rather than diagnosis and studies that were meta-analyses but were not, on closer inspection, concerned with diagnostic accuracy measures. Any discrepancies were resolved by mutual agreement.

Citation counts were retrieved on the 2 April 2020 from Scopus using a custom Python script developed and implemented by one of the authors, utilising the Scopus Application Programming Interface (Elsevier). The 100 top-cited meta-analyses were then identified from these retrieved citation counts and selected for further analysis. While meta-analyses were restricted to those published in radiology and medical imaging journals, citations originated from the wider medical literature in order to broadly capture the influence of these studies. The following information was extracted from these articles: (1) journal, (2) year of publication, (3) number of authors, (4) first and corresponding author, (5) institutional affiliations, (6) country of origin. The abstracts and/or full-text articles were also analysed to determine the imaging modalities reported, the anatomical region of interest and whether or not diagnostic accuracy was compared between modalities. The impact factor of individual journals was retrieved from the 2018 edition of *Journal Citation Reports* [10].

### Data analysis

Descriptive statistics were used to analyse the data. No statistical tests were performed. Data analysis was carried out using MATLAB 2019b (The MathWorks Inc).

## Results

**Fig. 1:**
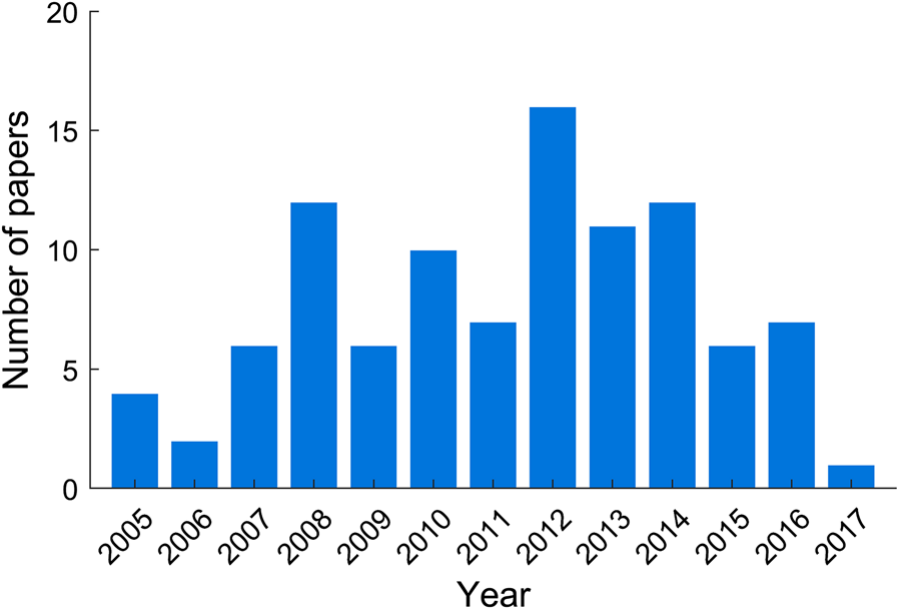
Number of top-cited meta-analyses by publication year. Histogram showing the number of top 100 meta-analyses published each year

The search yielded 1075 results. Of these, the top meta-analysis was cited 394 times, the 100th meta-analysis 38 times. The mean (median) number of citations for the 100 top-cited meta-analyses was 90.1 (62.5). The full list of meta-analyses is given in Additional file 1: Table S1. Although the search criteria allowed for meta-analyses published up to the end of 2019, no papers published in 2018 or 2019 featured in the top 100. The publication year with the most top meta-analyses was 2012 (16), followed by 2008 and 2014 (12 each) (Fig. 1).

Each paper had an average of 5.4 authors (minimum one, maximum 25). There were 17 authors with three or more meta-analyses in the top 100. The authors with the most citations were Bipat and Stoker who were associated with nine and seven papers, and a total of 1633 and 1595 citations, respectively (Table 1). Seven of these papers were in the top 10 most cited meta-analyses and were concerned predominantly with imaging of gastro-intestinal malignancies (colorectal cancer and pancreatic adenocarcinoma) and common acute gastro-intestinal inflammatory conditions (appendicitis, cholecystitis and diverticulitis).

**Table 1:** Authors with three or more top-cited meta-analysis papers

Rank	Author	Number of papers	Total citations
1	Bipat S	9	1633
2	Stoker J	7	1595
3	Dwamena BA	6	566
4	Hamon M	6	660
5	Chen JH	4	224
6	Kao CH	4	224
7	Sun Z	4	335
8	Boermeester MA	3	468
9	Bossuyt PM	3	439
10	Carlos RC	3	239
11	Lin WY	3	182
12	Lu YY	3	182
13	Morello R	3	330
14	Oudkerk M	3	241
15	Riddell JW	3	330
16	Yang J	3	162
17	Yuan L	3	162

A total of 254 affiliations were retrieved. There were 188 institutions represented (Table 2). Among these, the University of Amsterdam was represented the most times (10 papers), followed by China Medical University Hospital Taichung (seven papers), the University of Michigan Medical School (five papers) and VA Ann Arbor Healthcare System (five papers). These papers were again predominantly concerned with imaging of gastrointestinal malignancies and inflammatory conditions as well as urological malignancies, endocrine organ malignancies and ischaemic heart disease. In total, 23 countries were uniquely represented in a meta-analysis (Table 3). The USA appeared in 33 papers, followed by China and The Netherlands (both 17 papers), and the UK (10 papers).

**Table 2:** Institutions represented by at least three top-cited meta-analysis papers

Rank	Institution	Country	Number of papers
1	University of Amsterdam	The Netherlands	10
2	China Medical University Hospital Taichung	Taiwan	7
3	University of Michigan Medical School	United States	5
4	VA Ann Arbor Healthcare System	United States	5
5	Curtin University	Australia	4
6	Albert Einstein College of Medicine	United States	3
7	Beijing Friendship Hospital, Capital Medical University	China	3
8	Centre Hospitalier Universitaire de Caen Normandie	France	3
9	University Hospital Maastricht	The Netherlands	3
10	University Medical Center Groningen	The Netherlands	3

**Table 3:** Countries represented by top-cited meta-analysis papers

Rank	Country	Number of papers
1	United States	33
2	The Netherlands	18
3	China	17
4	Germany	9
5	United Kingdom	9
6	Canada	6
7	Italy	6
8	Taiwan	6
9	Australia	5
10	France	5
11	Japan	5
12	Denmark	3
13	Greece	2
14	Switzerland	2
15	Austria	1
16	Brazil	1
17	Finland	1
18	Iran	1
19	Israel	1
20	Malaysia	1
21	Pakistan	1
22	Singapore	1
23	South Korea	1

The 100 top-cited meta-analyses were published across 28 different journals. Among these, only 15 published two or more 100 top-cited meta-analyses (Table 4). The journal Radiology featured the most papers, with 24. This was also the journal with the highest impact factor (7.608).

**Table 4:** Journals that published at least two top-cited meta-analysis papers

Rank	Journal	Number of papers	Impact factor (2018)
1	Radiology	24	7.608
2	European Radiology	12	3.962
3	European Journal of Radiology	10	2.948
4	American Journal of Roentgenology	7	3.161
5	Journal of Nuclear Medicine	5	7.308
6	Nuclear Medicine Communications	5	1.465
7	Acta Radiologica	4	1.586
8	American Journal of Neuroradiology	3	3.256
9	Academic Radiology	3	2.267
10	Circulation: Cardiovascular Imaging	3	5.813
11	Journal of Ultrasound in Medicine	3	1.718
12	Clinical Radiology	2	2.082
13	Journal of Magnetic Resonance Imaging	2	3.732
14	Pediatric Radiology	2	2.022
15	Skeletal Radiology	2	1.518

**Fig. 2:**
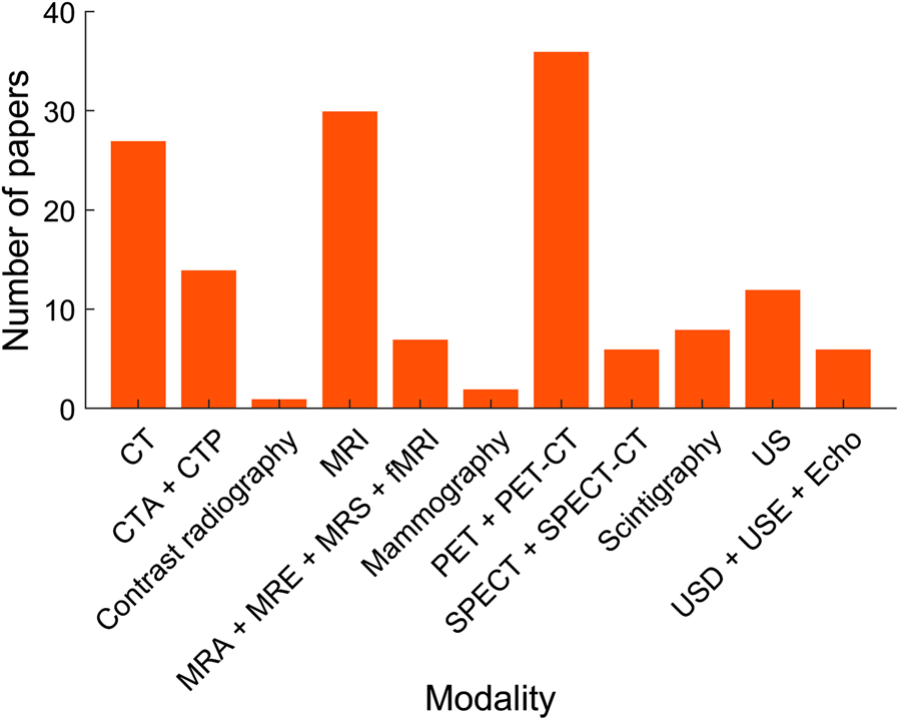
Imaging modalities represented in top-cited meta-analyses. Histogram showing the number of meta-analyses each imaging modality appears in. CT, computed tomography; CTA, CT angiography; MR, magnetic resonance; MRI, MR imaging; MRA, MR angiography; MRE, MR elastography; MRS, MR spectroscopy; fMRI, functional MRI; PET, positron emission tomography; SPECT, single-photon emission CT; US, ultrasound; USD, Doppler US; USE, US elastography; Echo, echocardiography

A broad range of imaging modalities were investigated in the 100 top-cited meta-analyses (Fig. 2). The most common modality reported was positron emission tomography (PET) or PET computed tomography (PET-CT), with 36 meta-analyses. Meta-analyses considering PET and/or PET-CT were concerned predominantly with cancer diagnosis and metastatic disease detection with prostate cancer being the most common, followed by colorectal cancer and lymphoma staging. The next most common imaging modality was magnetic resonance imaging (MRI) with 30 meta-analyses and computed tomography (CT) with 27 meta-analyses in which the most common conditions considered were prostate cancer, colorectal cancer and breast cancer for MRI and colorectal cancer and coronary artery disease for CT. Contrast radiography was the least common modality considered and was reported in a single study.

**Fig. 3:**
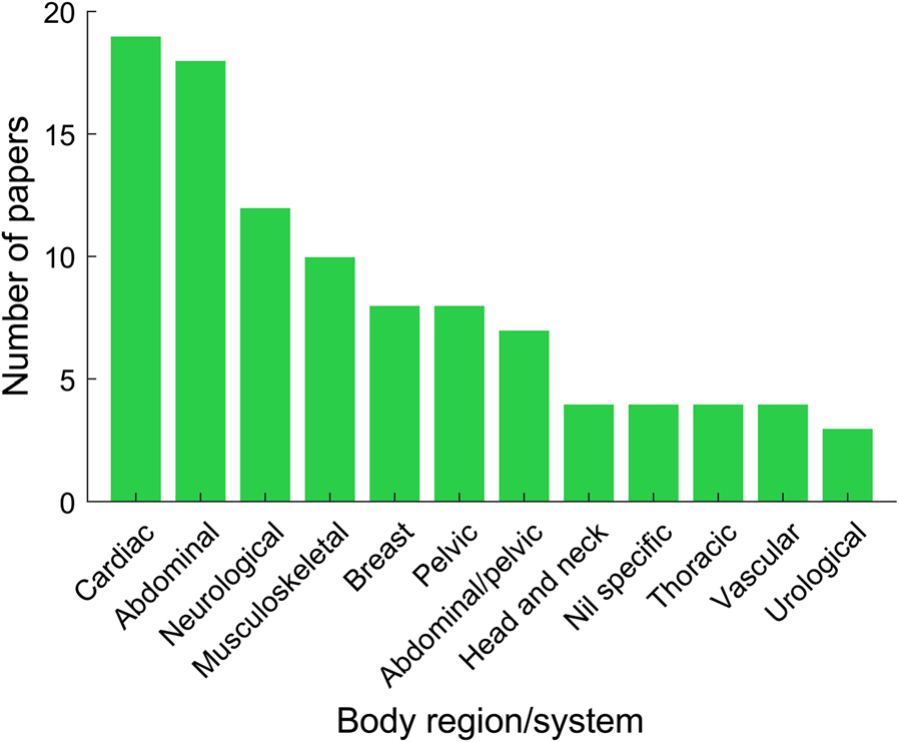
Body regions and organ systems represented in top-cited meta-analyses. Histogram showing the number of meta-analyses each body system appears in

Body region classification for each study was based on the region of the body deemed most pertinent to the organ or disease process being considered. Where no specific region was identifiable (for instance, neuroendocrine tumours or pyrexia of unknown origin), a classification of ‘General interest’ was assigned. Cardiac (19 papers), abdominal (18 papers), followed by neurological (12 papers) studies were the most frequently encountered in the 100 top-cited meta-analyses (Fig. 3).

We also found that 46% of the 100 top-cited meta-analyses were comparative diagnostic accuracy studies in which the diagnostic performance of one modality or imaging parameter was compared with one or more alternative modalities or imaging parameter (all against a common reference standard).

## Discussion

Many meta-analyses have investigated the diagnostic accuracy of common imaging modalities in a broad variety of conditions and body systems. In our study of the 100 top-cited meta-analyses, we found that the key imaging studies relate to cardiac, abdominal or neurological investigations. Furthermore, PET, PET-CT and MRI dominated the imaging modalities utilised. These findings attest to the importance of PET-CT in clinical oncology and research and of MRI in general. The interest in PET and PET-CT, in particular, is likely to be multifactorial and reflective of the increased use of PET for cancer staging [12], technological advancements in PET imaging and the development of new radiotracers as well as increasing interest in the clinical utility of hybrid and molecular imaging techniques [13, 14].

While it was unsurprising that studies focusing on cancer diagnosis and staging had the highest citation counts, it was interesting to note a lack of studies associated with lung cancer imaging. This could be reflective of peaking incidence rates of lung cancer in some Western countries due to tobacco control measures [15] and relatively less interest in new diagnostic tests compared with other malignancies (although we note the increasing interest in lung cancer screening using technologies such as low-dose CT [16]).

The most commonly cited studies had years of publication that appear to cluster around the period from 2008 to 2014. This may point towards an increase in interest towards diagnostic-accuracy-based meta-analyses in the field of radiology in these years or increasing confidence in the statistical techniques used in such studies (e.g. hierarchical models, which are more complex than methods used in meta-analyses for therapeutic interventions and increasingly used and recommended [17]). More recent studies may have lower citation counts as they have had less time to be of influence.

We also found that less than half of the meta-analyses compared one imaging modality against another. While such comparative analyses may be more useful in assessing new imaging tests against established modalities and diagnostic pathways, this suggests researchers may be more interested in studies of single modalities. This may reflect the fact that meta-analyses of diagnostic test accuracy, in general, often focus only on a single test and those which do address comparative test accuracy may be poorly designed or biased and difficult to interpret [18]

To our knowledge, this is the first bibliometric analysis of meta-analyses concerning diagnostic accuracy in radiology. In contrast, bibliometric studies of the general radiology literature are plentiful, for example [19–21]. In these studies, citation counts for the 100 top-cited articles ranged from 624 to 6447 [21], 371 to 6931 [19] and 422 to 7506 [20]. Even ignoring differences in publication dates, given that the top-cited meta-analysis was associated with 394 citations [22], meta-analyses of diagnostic accuracy are unlikely to feature substantially in these, or future bibliometric studies of the broader radiology literature. Our study may therefore provide a potential resource for identifying key diagnostic accuracy information related to imaging modalities used in the field of radiology which may be of particular interest to researchers, academics and practising radiologists with an interest in evidence-based diagnosis.

Although there are some differences between databases that report citation counts such as Web of Science, Scopus and Google Scholar [23], we elected to use Scopus due to the availability of an Application Programming Interface (API). This allowed efficient and complete extraction of author names, affiliations, publication details and citation metrics. One particular advantage of the Scopus database is the Affiliation Identifier that assigns each institution a unique number, thereby enabling aggregation of multiple affiliations from the same institution.

A key focus of bibliometric analysis is citation counts. Importantly, citation counts are influenced by a multitude of factors [24]. Some of these factors such as structured abstracts [25] and study design [5] may reflect the quality of reporting and strength of evidence. In contrast, other factors such as open access [26–28], and title length [8] may instead be related to visibility and accessibility. The association between citation counts and quality therefore needs to be considered carefully, especially given the risk of citation bias [6, 7], the preferential citing of statistically significant results that may lead to inflated expectations of efficacy. Certain studies may also be preferentially cited based on the reputations of the journals they are published in, even if they may have comparable quality to those published in lesser known journals. As this was a bibliometric study, we did not review the quality and reporting of meta-analyses and these considerations have been addressed elsewhere [2, 29–31]. The recently published guideline for reporting meta-analyses of diagnostic test accuracy, the PRISMA-DTA statement [32], provides a 27-item checklist for reporting such studies. Compliance with this reporting framework may be the subject of future investigation.

A related issue is that while citation counts, in this instance, may be a useful metric for gauging the extent to which the research community is interested in particular imaging modalities, they do not necessarily reflect clinical outcomes of these modalities or the feasibility of adopting new imaging technologies in clinical practice. This is especially relevant to meta-analyses of diagnostic accuracy which invariably focus on technical measures of diagnostic performance and not on parameters that may be of more clinical interest such as cost-effectiveness, ease of access or patient and clinician acceptability.

Our study had some limitations. First, the Scopus API was the primary tool used for extracting citation counts, authorship, and affiliations. We noted that the affiliations recorded in the retrieved document and affiliation profile were an aggregate of those listed in the published articles. While such an approach prevented the discrimination between first and corresponding author affiliations, it instead afforded the opportunity to capture all distinct institutional affiliations. Thus, in our analysis we did not distinguish between the affiliations of different authors. Second, our search was not limited to radiology-specific journals in the category “Radiology, Nuclear Medicine and Imaging” by *Journal Citation Reports* (Clarivate Analytics, 2018) [10]. Instead, we included every journal in the category. Notably, despite conducting a broad search, the meta-analyses retrieved were predominantly published in radiology journals, whereas only several papers were published in cardiovascular imaging or nuclear medicine journals. However, by restricting the search to a single category, relevant meta-analyses published in other general medicine or specialty journals may not have been included. Third, differences in citations may exist between different search databases [23]. We mitigate this by including an extensive list of meta-analyses, that is of the 100 top-cited papers. Fourth, we restricted our search to meta-analyses due to the fact that these provide concise and readily interpretable summary information of a quantitative nature. However, both systematic reviews and meta-analyses are widely considered to sit at the top of the hierarchy of evidence. While many meta-analyses incorporate a systematic review in order to identify all relevant primary studies, not all systematic reviews incorporate meta-analysis (due to lack of primary studies or significant underlying heterogeneity). Therefore, it is possible that important evidence syntheses may not have been identified by our current work.

There are newer means of assessment of the influence of scientific publications such as Altmetrics which provide information on citations in the broader online and media community and may be complementary to traditional, citation-based metrics. While it is presumed that imaging-based diagnostic accuracy meta-analyses might receive less attention in these domains due to their technical nature, some studies of influential imaging modalities may receive wide attention in the media and this impact has not been evaluated here.

In conclusion, our bibliometric study provides a collation of the most influential meta-analyses of diagnostic accuracy in radiology as measured by citation counts. The 100 top-cited meta-analyses encompass a broad range of imaging modalities and body regions. However, we emphasise that citation count and quality should not be confounded, and that individual papers be considered on their own merits.

## Supplementary information

**Additional file 1:** PubMed search string and full list of meta-analyses.

## Data Availability

All data analysed in this study is available in the main text or supplementary section.

## Abbreviation

API: Application Programming Interface
