# Supplementary material for: The 100 top-cited meta-analyses of diagnostic accuracy in radiology journals: a bibliometric analysis
Source: Insights Imaging. 2020 Nov 23;11:123. doi: 10.1186/s13244-020-00936-w (PMC7683640; doi:10.1186/s13244-020-00936-w)
Supplement: Supplementary file 1 — Additional file 1: PubMed search string and full list of meta-analyses. [file 13244_2020_936_MOESM1_ESM.docx]

**Electronic Supplementary Material**

**SUPPLEMENTARY A**

The PubMed search string used is given below.

((meta-analysis[Title/Abstract]) OR (meta-analysis as topic[MeSH Terms]) OR(meta-analysis[Publication Type])) AND ((JACC-Cardiovascular Imaging[Journal]) OR (MEDICAL IMAGE ANALYSIS[Journal]) OR (IEEE TRANSACTIONS ON MEDICAL IMAGING[Journal]) OR (RADIOLOGY[Journal]) OR (JOURNAL OF NUCLEAR MEDICINE[Journal]) OR (EUROPEAN JOURNAL OF NUCLEAR MEDICINE AND MOLECULAR IMAGING[Journal]) OR (CLINICAL NUCLEAR MEDICINE[Journal]) OR (INTERNATIONAL JOURNAL OF RADIATION ONCOLOGY BIOLOGY PHYSICS[Journal]) OR (INVESTIGATIVE RADIOLOGY[Journal]) OR (Circulation-Cardiovascular Imaging[Journal]) OR (NEUROIMAGE[Journal]) OR (ULTRASOUND IN OBSTETRICS & GYNECOLOGY[Journal]) OR (European Heart Journal-Cardiovascular Imaging[Journal]) OR (RADIOTHERAPY AND ONCOLOGY[Journal]) OR (Photoacoustics[Journal]) OR (JOURNAL OF CARDIOVASCULAR MAGNETIC RESONANCE[Journal]) OR (ULTRASCHALL IN DER MEDIZIN[Journal]) OR (HUMAN BRAIN MAPPING[Journal]) OR (JOURNAL OF NUCLEAR CARDIOLOGY[Journal]) OR (EUROPEAN RADIOLOGY[Journal]) OR (RADIOGRAPHICS[Journal]) OR (Biomedical Optics Express[Journal]) OR (MAGNETIC RESONANCE IN MEDICINE[Journal]) OR (SEMINARS IN NUCLEAR MEDICINE[Journal]) OR (Journal of the American College of Radiology[Journal]) OR (JOURNAL OF MAGNETIC RESONANCE IMAGING[Journal]) OR (KOREAN JOURNAL OF RADIOLOGY[Journal]) OR (INTERNATIONAL JOURNAL OF HYPERTHERMIA[Journal]) OR (EJNMMI Physics[Journal]) OR (NMR IN BIOMEDICINE[Journal]) OR (MOLECULAR IMAGING AND BIOLOGY[Journal]) OR (Journal of Cardiovascular Computed Tomography[Journal]) OR (COMPUTERIZED MEDICAL IMAGING AND GRAPHICS[Journal]) OR (AMERICAN JOURNAL OF NEURORADIOLOGY[Journal]) OR (MEDICAL PHYSICS[Journal]) OR (AMERICAN JOURNAL OF ROENTGENOLOGY[Journal]) OR (CANCER IMAGING[Journal]) OR (Quantitative Imaging in Medicine and Surgery[Journal]) OR (PHYSICS IN MEDICINE AND BIOLOGY[Journal]) OR (EJNMMI Research[Journal]) OR (EUROPEAN JOURNAL OF RADIOLOGY[Journal]) OR (Radiation Oncology[Journal]) OR (MAGNETIC RESONANCE MATERIALS IN PHYSICS BIOLOGY AND MEDICINE[Journal]) OR (JOURNAL OF VASCULAR AND INTERVENTIONAL RADIOLOGY[Journal]) OR (Clinical Neuroradiology[Journal]) OR (Practical Radiation Oncology[Journal]) OR (RADIATION RESEARCH[Journal]) OR (SEMINARS IN RADIATION ONCOLOGY[Journal]) OR (STRAHLENTHERAPIE UND ONKOLOGIE[Journal]) OR (ULTRASONICS[Journal]) OR (JOURNAL OF DIGITAL IMAGING[Journal]) OR (JOURNAL OF BIOMEDICAL OPTICS[Journal]) OR (Physica Medica-European Journal of Medical Physics[Journal]) OR (NEURORADIOLOGY[Journal]) OR (NUCLEAR MEDICINE AND BIOLOGY[Journal]) OR (ULTRASONIC IMAGING[Journal]) OR (Diagnostic and Interventional Imaging[Journal]) OR (JOURNAL OF NEURORADIOLOGY[Journal]) OR (Dose-Response[Journal]) OR (Zeitschrift fur Medizinische Physik[Journal]) OR (ACADEMIC RADIOLOGY[Journal]) OR (INTERNATIONAL JOURNAL OF RADIATION BIOLOGY[Journal]) OR (ULTRASOUND IN MEDICINE AND BIOLOGY[Journal]) OR (International Journal of Computer Assisted Radiology and Surgery[Journal]) OR (Abdominal Radiology[Journal]) OR (MAGNETIC RESONANCE IMAGING[Journal]) OR (CLINICAL RADIOLOGY[Journal]) OR (JOURNAL OF NEUROIMAGING[Journal]) OR (JOURNAL OF THORACIC IMAGING[Journal]) OR (NEUROIMAGING CLINICS OF NORTH AMERICA[Journal]) OR (Brachytherapy[Journal]) OR (PEDIATRIC RADIOLOGY[Journal]) OR (JOURNAL OF RADIATION RESEARCH[Journal]) OR (Magnetic Resonance Imaging Clinics of North America[Journal]) OR (Contrast Media & Molecular Imaging[Journal]) OR (Molecular Imaging[Journal]) OR (BRITISH JOURNAL OF RADIOLOGY[Journal]) OR (QUARTERLY JOURNAL OF NUCLEAR MEDICINE AND MOLECULAR IMAGING[Journal]) OR (CARDIOVASCULAR AND INTERVENTIONAL RADIOLOGY[Journal]) OR (CANCER BIOTHERAPY AND RADIOPHARMACEUTICALS[Journal]) OR (ROFO-FORTSCHRITTE AUF DEM GEBIET DER RONTGENSTRAHLEN UND DER BILDGEBENDEN VERFAHREN[Journal]) OR (RADIOLOGIC CLINICS OF NORTH AMERICA[Journal]) OR (INTERNATIONAL JOURNAL OF CARDIOVASCULAR IMAGING[Journal]) OR (Journal of Contemporary Brachytherapy[Journal]) OR (Radiology and Oncology[Journal]) OR (JOURNAL OF ULTRASOUND IN MEDICINE[Journal]) OR (Medical Ultrasonography[Journal]) OR (ANNALS OF NUCLEAR MEDICINE[Journal]) OR (BMC MEDICAL IMAGING[Journal]) OR (ACTA RADIOLOGICA[Journal]) OR (Journal of Applied Clinical Medical Physics[Journal]) OR (DENTOMAXILLOFACIAL RADIOLOGY[Journal]) OR (SKELETAL RADIOLOGY[Journal]) OR (Japanese Journal of Radiology[Journal]) OR (Magnetic Resonance in Medical Sciences[Journal]) OR (NUCLEAR MEDICINE COMMUNICATIONS[Journal]) OR (Diagnostic and Interventional Radiology[Journal]) OR (INTERVENTIONAL NEURORADIOLOGY[Journal]) OR (Radiologia Medica[Journal]) OR (APPLIED RADIATION AND ISOTOPES[Journal]) OR (JOURNAL OF RADIOLOGICAL PROTECTION[Journal]) OR (JOURNAL OF COMPUTER ASSISTED TOMOGRAPHY[Journal]) OR (NUKLEARMEDIZIN-NUCLEAR MEDICINE[Journal]) OR (RADIATION AND ENVIRONMENTAL BIOPHYSICS[Journal]) OR (Cancer Radiotherapie[Journal]) OR (Journal of Medical Imaging and Radiation Oncology[Journal]) OR (SEMINARS IN ULTRASOUND CT AND MRI[Journal]) OR (SEMINARS IN MUSCULOSKELETAL RADIOLOGY[Journal]) OR (CLINICAL IMAGING[Journal]) OR (Medical Dosimetry[Journal]) OR (Journal of Innovative Optical Health Sciences[Journal]) OR (SURGICAL AND RADIOLOGIC ANATOMY[Journal]) OR (CANADIAN ASSOCIATION OF RADIOLOGISTS JOURNAL-JOURNAL DE L ASSOCIATION CANADIENNE DES RADIOLOGISTES[Journal]) OR (HEALTH PHYSICS[Journal]) OR (SEMINARS IN INTERVENTIONAL RADIOLOGY[Journal]) OR (Journal of Medical Ultrasonics[Journal]) OR (Hellenic Journal of Nuclear Medicine[Journal]) OR (Revista Espanola de Medicina Nuclear e Imagen Molecular[Journal]) OR (Ultrasound Quarterly[Journal]) OR (RADIATION PROTECTION DOSIMETRY[Journal]) OR (JOURNAL OF CLINICAL ULTRASOUND[Journal]) OR (SEMINARS IN ROENTGENOLOGY[Journal]) OR (Current Medical Imaging Reviews[Journal]) OR (International Journal of Radiation Research[Journal]) OR (Journal of Medical Imaging and Health Informatics[Journal]) OR (Iranian Journal of Radiology[Journal]) OR (Journal of the Belgian Society of Radiology[Journal]) OR (RADIOPROTECTION[Journal]) OR (RADIOLOGE[Journal]))((diagnostic test accuracy OR DTA [all fields]) OR (sensitivity and specificity [all fields]) OR (sensitiv* [all fields]) OR (specificit* [all fields]) OR (accuracy [all fields]) OR  (ROC [all fields]) OR (receiver operator characteristic [all fields]) OR (odds ratio [all fields]) OR (likelihood ratio [all fields]) OR (positive predictive value[all fields]) OR (PPV[all fields]) OR (negative predictive value[all fields]) OR (NPV[all fields]) OR (false positive* [all fields]) OR (false negative* [all fields])) AND (2005/01/01[PDat]: 2019/12/31[PDat])

**SUPPLEMENTARY B**

**Supplementary Table 1. Top 100 most-cited meta-analyses of diagnostic accuracy in radiology.**

| Rank | Title | Modalities | Regions/systems | Comparative | Citations |
| --- | --- | --- | --- | --- | --- |
| 1 | Horsthuis K, Bipat S, Bennink RJ, Stoker J. Inflammatory bowel disease diagnosed with US, MR, scintigraphy, and CT: meta-analysis of prospective studies. Radiology. 2008;247(1):64-79 | US, MRI, CT, PET, scintigraphy | Abdominal/pelvic | Yes | 394 |
| 2 | Niekel MC, Bipat S, Stoker J. Diagnostic imaging of colorectal liver metastases with CT, MR imaging, FDG PET, and/or FDG PET/CT: a meta-analysis of prospective studies including patients who have not previously undergone treatment. Radiology. 2010;257(3):674-84. | CT, MRI, PET, PET-CT | Abdominal | Yes | 315 |
| 3 | de Jesus JO, Parker L, Frangos AJ, Nazarian LN. Accuracy of MRI, MR arthrography, and ultrasound in the diagnosis of rotator cuff tears: a meta-analysis. AJR Am J Roentgenol. 2009;192(6):1701-7. | MRI, US | Musculoskeletal | Yes | 310 |
| 4 | Youssef G, Leung E, Mylonas I, Nery P, Williams K, Wisenberg G, et al. The use of 18F-FDG PET in the diagnosis of cardiac sarcoidosis: a systematic review and metaanalysis including the Ontario experience. J Nucl Med. 2012;53(2):241-8. | PET | Cardiac | No | 266 |
| 5 | Pickhardt PJ, Hassan C, Halligan S, Marmo R. Colorectal cancer: CT colonography and colonoscopy for detection--systematic review and meta-analysis. Radiology. 2011;259(2):393-405. | CT | Abdominal/pelvic | Yes | 251 |
| 6 | Pakos EE, Fotopoulos AD, Ioannidis JP. 18F-FDG PET for evaluation of bone marrow infiltration in staging of lymphoma: a meta-analysis. J Nucl Med. 2005;46(6):958-63. | PET | Musculoskeletal | No | 215 |
| 7 | van Randen A, Bipat S, Zwinderman AH, Ubbink DT, Stoker J, Boermeester MA. Acute appendicitis: meta-analysis of diagnostic performance of CT and graded compression US related to prevalence of disease. Radiology. 2008;249(1):97-106. | US, CT | Abdominal/pelvic | Yes | 203 |
| 8 | van der Paardt MP, Zagers MB, Beets-Tan RG, Stoker J, Bipat S. Patients who undergo preoperative chemoradiotherapy for locally advanced rectal cancer restaged by using diagnostic MR imaging: a systematic review and meta-analysis. Radiology. 2013;269(1):101-12. | MRI | Abdominal/pelvic | No | 190 |
| 9 | Hamon M, Morello R, Riddell JW, Hamon M. Coronary arteries: diagnostic performance of 16- versus 64-section spiral CT compared with invasive coronary angiography--meta-analysis. Radiology. 2007;245(3):720-31. | CT | Cardiac | Yes | 182 |
| 10 | Bipat S, Phoa SS, van Delden OM, Bossuyt PM, Gouma DJ, Laméris JS, et al. Ultrasonography, computed tomography and magnetic resonance imaging for diagnosis and determining resectability of pancreatic adenocarcinoma: a meta-analysis. J Comput Assist Tomogr. 2005;29(4):438-45. | US, CT, MRI | Abdominal | Yes | 174 |
| 11 | Niemann T, Kollmann T, Bongartz G. Diagnostic performance of low-dose CT for the detection of urolithiasis: a meta-analysis. AJR Am J Roentgenol. 2008;191(2):396-401. | CT | Urological | No | 172 |
| 12 | Floriani I, Torri V, Rulli E, Garavaglia D, Compagnoni A, Salvolini L, et al. Performance of imaging modalities in diagnosis of liver metastases from colorectal cancer: a systematic review and meta-analysis. J Magn Reson Imaging. 2010;31(1):19-31. | MRI, CT, PET, US | Abdominal | Yes | 156 |
| 13 | Takx RA, Blomberg BA, El Aidi H, Habets J, de Jong PA, Nagel E, et al. Diagnostic accuracy of stress myocardial perfusion imaging compared to invasive coronary angiography with fractional flow reserve meta-analysis. Circ Cardiovasc Imaging. 2015;8(1). | SPECT, echo, MRI, PET, CT | Cardiac | Yes | 145 |
| 14 | Cronin P, Dwamena BA, Kelly AM, Carlos RC. Solitary pulmonary nodules: meta-analytic comparison of cross-sectional imaging modalities for diagnosis of malignancy. Radiology. 2008;246(3):772-82. | CT, MRI, PET, SPECT | Thoracic | Yes | 145 |
| 15 | Dave M, Elmunzer BJ, Dwamena BA, Higgins PD. Primary sclerosing cholangitis: meta-analysis of diagnostic performance of MR cholangiopancreatography. Radiology. 2010;256(2):387-96. | MRI | Abdominal | No | 142 |
| 16 | Menke J. Diagnostic accuracy of multidetector CT in acute mesenteric ischemia: systematic review and meta-analysis. Radiology. 2010;256(1):93-101. | CT | Abdominal | No | 142 |
| 17 | Laméris W, van Randen A, Bipat S, Bossuyt PM, Boermeester MA, Stoker J. Graded compression ultrasonography and computed tomography in acute colonic diverticulitis: meta-analysis of test accuracy. Eur Radiol. 2008;18(11):2498-511. | US, CT | Abdominal | Yes | 142 |
| 18 | Dunet V, Rossier C, Buck A, Stupp R, Prior JO. Performance of 18F-fluoro-ethyl-tyrosine (18F-FET) PET for the differential diagnosis of primary brain tumor: a systematic review and Metaanalysis. J Nucl Med. 2012;53(2):207-14. | PET | Neurological | No | 141 |
| 19 | Boland GW, Dwamena BA, Jagtiani Sangwaiya M, Goehler AG, Blake MA, Hahn PF, et al. Characterization of adrenal masses by using FDG PET: a systematic review and meta-analysis of diagnostic test performance. Radiology. 2011;259(1):117-26. | PET | Abdominal | No | 140 |
| 20 | Shen G, Deng H, Hu S, Jia Z. Comparison of choline-PET/CT, MRI, SPECT, and bone scintigraphy in the diagnosis of bone metastases in patients with prostate cancer: a meta-analysis. Skeletal Radiol. 2014;43(11):1503-13. | PET-CT, SPECT, scintigraphy | Musculoskeletal | Yes | 137 |
| 21 | Westerlaan HE, van Dijk JM, Jansen-van der Weide MC, de Groot JC, Groen RJ, Mooij JJ, et al. Intracranial aneurysms in patients with subarachnoid hemorrhage: CT angiography as a primary examination tool for diagnosis--systematic review and meta-analysis. Radiology. 2011;258(1):134-45. | CT | Neurological | No | 135 |
| 22 | Romero J, Husain SA, Kelesidis I, Sanz J, Medina HM, Garcia MJ. Detection of left atrial appendage thrombus by cardiac computed tomography in patients with atrial fibrillation: a meta-analysis. Circ Cardiovasc Imaging. 2013;6(2):185-94. | CT, echo | Cardiac | Yes | 130 |
| 23 | Sun Z, Lin C, Davidson R, Dong C, Liao Y. Diagnostic value of 64-slice CT angiography in coronary artery disease: a systematic review. Eur J Radiol. 2008;67(1):78-84. | CT | Cardiac | No | 124 |
| 24 | Kiewiet JJ, Leeuwenburgh MM, Bipat S, Bossuyt PM, Stoker J, Boermeester MA. A systematic review and meta-analysis of diagnostic performance of imaging in acute cholecystitis. Radiology. 2012;264(3):708-20. | Scintigraphy | Abdominal | Yes | 123 |
| 25 | Sun Z, Jiang W. Diagnostic value of multislice computed tomography angiography in coronary artery disease: a meta-analysis. Eur J Radiol. 2006;60(2):279-86. | CT | Cardiac | No | 121 |
| 26 | Kwee RM. Prediction of tumor response to neoadjuvant therapy in patients with esophageal cancer with use of 18F FDG PET: a systematic review. Radiology. 2010;254(3):707-17. | PET | Thoracic, abdominal | No | 103 |
| 27 | Tsushima Y, Takahashi-Taketomi A, Endo K. Magnetic resonance (MR) differential diagnosis of breast tumors using apparent diffusion coefficient (ADC) on 1.5-T. J Magn Reson Imaging. 2009;30(2):249-55. | MRI | Breast | No | 101 |
| 28 | Vinnicombe S, Pinto Pereira SM, McCormack VA, Shiel S, Perry N, Dos Santos Silva IM. Full-field digital versus screen-film mammography: comparison within the UK breast screening program and systematic review of published data. Radiology. 2009;251(2):347-58. | Mammography | Breast | Yes | 100 |
| 29 | Wu LM, Xu JR, Ye YQ, Lu Q, Hu JN. The clinical value of diffusion-weighted imaging in combination with T2-weighted imaging in diagnosing prostate carcinoma: a systematic review and meta-analysis. AJR Am J Roentgenol. 2012;199(1):103-10. | MRI | Pelvic | Yes | 98 |
| 30 | Romero J, Xue X, Gonzalez W, Garcia MJ. CMR imaging assessing viability in patients with chronic ventricular dysfunction due to coronary artery disease: a meta-analysis of prospective trials. JACC Cardiovasc Imaging. 2012;5(5):494-508. | MRI | Cardiac | Yes | 94 |
| 31 | Tan CH, Wei W, Johnson V, Kundra V. Diffusion-weighted MRI in the detection of prostate cancer: meta-analysis. AJR Am J Roentgenol. 2012;199(4):822-9. | MRI | Pelvic | Yes | 93 |
| 32 | Hamon M, Lepage O, Malagutti P, Riddell JW, Morello R, Agostini D, et al. Diagnostic performance of 16- and 64-section spiral CT for coronary artery bypass graft assessment: meta-analysis. Radiology. 2008;247(3):679-86. | CT | Cardiac | Yes | 92 |
| 33 | Smith TO, Back T, Toms AP, Hing CB. Diagnostic accuracy of ultrasound for rotator cuff tears in adults: a systematic review and meta-analysis. Clin Radiol. 2011;66(11):1036-48. | US | Musculoskeletal | Yes | 91 |
| 34 | Heijenbrok-Kal MH, Kock MC, Hunink MG. Lower extremity arterial disease: multidetector CT angiography meta-analysis. Radiology. 2007;245(2):433-9. | CT | Vascular | No | 88 |
| 35 | von Eyben FE, Kairemo K. Meta-analysis of (11)C-choline and (18)F-choline PET/CT for management of patients with prostate cancer. Nucl Med Commun. 2014;35(3):221-30. | PET-CT | Pelvic | No | 88 |
| 36 | Dym RJ, Burns J, Freeman K, Lipton ML. Is functional MR imaging assessment of hemispheric language dominance as good as the Wada test?: a meta-analysis. Radiology. 2011;261(2):446-55. | fMRI | Neurological | No | 82 |
| 37 | Baltzer PA, Dietzel M. Breast lesions: diagnosis by using proton MR spectroscopy at 1.5 and 3.0 T--systematic review and meta-analysis. Radiology. 2013;267(3):735-46. | MR-spectroscopy | Breast | No | 80 |
| 38 | Hayashino Y, Goto M, Noguchi Y, Fukui T. Ventilation-perfusion scanning and helical CT in suspected pulmonary embolism: meta-analysis of diagnostic performance. Radiology. 2005;234(3):740-8. | CT, scintigraphy | Thoracic | Yes | 80 |
| 39 | Deppen SA, Blume J, Bobbey AJ, Shah C, Graham MM, Lee P, et al. 68Ga-DOTATATE Compared with 111In-DTPA-Octreotide and Conventional Imaging for Pulmonary and Gastroenteropancreatic Neuroendocrine Tumors: A Systematic Review and Meta-Analysis. J Nucl Med. 2016;57(6):872-8. | PET-CT | General interest | No | 79 |
| 40 | Nihashi T, Dahabreh IJ, Terasawa T. Diagnostic accuracy of PET for recurrent glioma diagnosis: a meta-analysis. AJNR Am J Neuroradiol. 2013;34(5):944-50, s1-11. | PET | Neurological | No | 76 |
| 41 | Singh S, Venkatesh SK, Loomba R, Wang Z, Sirlin C, Chen J, et al. Magnetic resonance elastography for staging liver fibrosis in non-alcoholic fatty liver disease: a diagnostic accuracy systematic review and individual participant data pooled analysis. Eur Radiol. 2016;26(5):1431-40. | MR-elastography | Abdominal | No | 76 |
| 42 | Yuan Y, Chen XS, Liu SY, Shen KW. Accuracy of MRI in prediction of pathologic complete remission in breast cancer after preoperative therapy: a meta-analysis. AJR Am J Roentgenol. 2010;195(1):260-8. | MRI | Breast | No | 74 |
| 43 | Prandini N, Lazzeri E, Rossi B, Erba P, Parisella MG, Signore A. Nuclear medicine imaging of bone infections. Nucl Med Commun. 2006;27(8):633-44. | PET, scintigraphy | Musculoskeletal | Yes | 71 |
| 44 | Lu YY, Chen JH, Liang JA, Wang HY, Lin CC, Lin WY, et al. Clinical value of FDG PET or PET/CT in urinary bladder cancer: a systemic review and meta-analysis. Eur J Radiol. 2012;81(9):2411-6. | PET, PET-CT | Urological | No | 70 |
| 45 | Yang J, Kan Y, Ge BH, Yuan L, Li C, Zhao W. Diagnostic role of Gallium-68 DOTATOC and Gallium-68 DOTATATE PET in patients with neuroendocrine tumors: a meta-analysis. Acta Radiol. 2014;55(4):389-98. | PET | General interest | Yes | 69 |
| 46 | Thomas SM, Goodacre SW, Sampson FC, van Beek EJ. Diagnostic value of CT for deep vein thrombosis: results of a systematic review and meta-analysis. Clin Radiol. 2008;63(3):299-304. | CT | Vascular | No | 68 |
| 47 | Wang HY, Ding HJ, Chen JH, Chao CH, Lu YY, Lin WY, et al. Meta-analysis of the diagnostic performance of [18F]FDG-PET and PET/CT in renal cell carcinoma. Cancer Imaging. 2012;12(3):464-74. | PET, PET-CT | Urological | Yes | 66 |
| 48 | Andreano A, Rechichi G, Rebora P, Sironi S, Valsecchi MG, Galimberti S. MR diffusion imaging for preoperative staging of myometrial invasion in patients with endometrial cancer: a systematic review and meta-analysis. Eur Radiol. 2014;24(6):1327-38. | MRI | Pelvic | Yes | 64 |
| 49 | Sosna J, Sella T, Sy O, Lavin PT, Eliahou R, Fraifeld S, et al. Critical analysis of the performance of double-contrast barium enema for detecting colorectal polyps > or = 6 mm in the era of CT colonography. AJR Am J Roentgenol. 2008;190(2):374-85. | Contrast-radiography, CT | Abdominal/pelvic | Yes | 63 |
| 50 | Tateishi U, Morita S, Taguri M, Shizukuishi K, Minamimoto R, Kawaguchi M, et al. A meta-analysis of (18)F-Fluoride positron emission tomography for assessment of metastatic bone tumor. Ann Nucl Med. 2010;24(7):523-31. | PET, PET-CT, SPECT, scintigraphy | Musculoskeletal | Yes | 63 |
| 51 | Kwee TC, Kwee RM. MR angiography in the follow-up of intracranial aneurysms treated with Guglielmi detachable coils: systematic review and meta-analysis. Neuroradiology. 2007;49(9):703-13. | MRA | Neurological | Yes | 62 |
| 52 | Hanna RF, Miloushev VZ, Tang A, Finklestone LA, Brejt SZ, Sandhu RS, et al. Comparative 13-year meta-analysis of the sensitivity and positive predictive value of ultrasound, CT, and MRI for detecting hepatocellular carcinoma. Abdom Radiol (NY). 2016;41(1):71-90. | MRI, CT, US | Abdominal | Yes | 62 |
| 53 | Chappell FM, Wardlaw JM, Young GR, Gillard JH, Roditi GH, Yip B, et al. Carotid artery stenosis: accuracy of noninvasive tests--individual patient data meta-analysis. Radiology. 2009;251(2):493-502. | Doppler-US, MRA, CTA | Vascular | Yes | 61 |
| 54 | Fischer C, Hulten E, Belur P, Smith R, Voros S, Villines TC. Coronary CT angiography versus intravascular ultrasound for estimation of coronary stenosis and atherosclerotic plaque burden: a meta-analysis. J Cardiovasc Comput Tomogr. 2013;7(4):256-66. | CTA | Cardiac | No | 60 |
| 55 | Dorrius MD, Dijkstra H, Oudkerk M, Sijens PE. Effect of b value and pre-admission of contrast on diagnostic accuracy of 1.5-T breast DWI: a systematic review and meta-analysis. Eur Radiol. 2014;24(11):2835-47. | MRI | Breast | Yes | 60 |
| 56 | Lin P, Chen M, Liu B, Wang S, Li X. Diagnostic performance of shear wave elastography in the identification of malignant thyroid nodules: a meta-analysis. Eur Radiol. 2014;24(11):2729-38. | US-elastography | Head and neck | No | 59 |
| 57 | Nielsen LH, Ortner N, Nørgaard BL, Achenbach S, Leipsic J, Abdulla J. The diagnostic accuracy and outcomes after coronary computed tomography angiography vs. conventional functional testing in patients with stable angina pectoris: a systematic review and meta-analysis. Eur Heart J Cardiovasc Imaging. 2014;15(9):961-71. | SPECT, CTA | Cardiac | Yes | 58 |
| 58 | Greenberg ED, Gold R, Reichman M, John M, Ivanidze J, Edwards AM, et al. Diagnostic accuracy of CT angiography and CT perfusion for cerebral vasospasm: a meta-analysis. AJNR Am J Neuroradiol. 2010;31(10):1853-60. | CTA, CTP | Neurological | Yes | 56 |
| 59 | Hamon M, Champ-Rigot L, Morello R, Riddell JW, Hamon M. Diagnostic accuracy of in-stent coronary restenosis detection with multislice spiral computed tomography: a meta-analysis. Eur Radiol. 2008;18(2):217-25. | CTA | Cardiac | No | 56 |
| 60 | Kang S, Kim SK, Chung DC, Seo SS, Kim JY, Nam BH, et al. Diagnostic value of (18)F-FDG PET for evaluation of paraaortic nodal metastasis in patients with cervical carcinoma: a metaanalysis. J Nucl Med. 2010;51(3):360-7. | PET | Abdominal | No | 56 |
| 61 | Sun J, Cai J, Wang X. Real-time ultrasound elastography for differentiation of benign and malignant thyroid nodules: a meta-analysis. J Ultrasound Med. 2014;33(3):495-502. | US-elastography | Head and neck | Yes | 55 |
| 62 | Foerster BR, Dwamena BA, Petrou M, Carlos RC, Callaghan BC, Churchill CL, et al. Diagnostic accuracy of diffusion tensor imaging in amyotrophic lateral sclerosis: a systematic review and individual patient data meta-analysis. Acad Radiol. 2013;20(9):1099-106. | MRI | Neurological | No | 55 |
| 63 | Ruys AT, van Beem BE, Engelbrecht MR, Bipat S, Stoker J, Van Gulik TM. Radiological staging in patients with hilar cholangiocarcinoma: a systematic review and meta-analysis. Br J Radiol. 2012;85(1017):1255-62. | CT | Abdominal | No | 54 |
| 64 | Stengel D, Bauwens K, Rademacher G, Mutze S, Ekkernkamp A. Association between compliance with methodological standards of diagnostic research and reported test accuracy: meta-analysis of focused assessment of US for trauma. Radiology. 2005;236(1):102-11. | US | Abdominal | No | 54 |
| 65 | Zhang H, Ma L, Wang Q, Zheng X, Wu C, Xu BN. Role of magnetic resonance spectroscopy for the differentiation of recurrent glioma from radiation necrosis: a systematic review and meta-analysis. Eur J Radiol. 2014;83(12):2181-9. | MR-spectroscopy | Neurological | No | 53 |
| 66 | Lei J, Yang P, Zhang L, Wang Y, Yang K. Diagnostic accuracy of digital breast tomosynthesis versus digital mammography for benign and malignant lesions in breasts: a meta-analysis. Eur Radiol. 2014;24(3):595-602. | Mammography | Breast | Yes | 53 |
| 67 | Pakos EE, Trikalinos TA, Fotopoulos AD, Ioannidis JP. Prosthesis infection: diagnosis after total joint arthroplasty with antigranulocyte scintigraphy with 99mTc-labeled monoclonal antibodies--a meta-analysis. Radiology. 2007;242(1):101-8. | Scintigraphy | Musculoskeletal | No | 50 |
| 68 | Ren J, Yuan L, Wen G, Yang J. The value of anti-1-amino-3-18F-fluorocyclobutane-1-carboxylic acid PET/CT in the diagnosis of recurrent prostate carcinoma: a meta-analysis. Acta Radiol. 2016;57(4):487-93. | PET-CT | Pelvic | No | 48 |
| 69 | Zhang L, Tang M, Min Z, Lu J, Lei X, Zhang X. Accuracy of combined dynamic contrast-enhanced magnetic resonance imaging and diffusion-weighted imaging for breast cancer detection: a meta-analysis. Acta Radiol. 2016;57(6):651-60. | MRI | Breast | Yes | 48 |
| 70 | Thomeer MG, Gerestein C, Spronk S, van Doorn HC, van der Ham E, Hunink MG. Clinical examination versus magnetic resonance imaging in the pretreatment staging of cervical carcinoma: systematic review and meta-analysis. Eur Radiol. 2013;23(7):2005-18. | Clinical exam, MRI | Pelvic | Yes | 48 |
| 71 | Samad Z, Hakeem A, Mahmood SS, Pieper K, Patel MR, Simel DL, et al. A meta-analysis and systematic review of computed tomography angiography as a diagnostic triage tool for patients with chest pain presenting to the emergency department. J Nucl Cardiol. 2012;19(2):364-76. | CTA | Cardiac | No | 47 |
| 72 | Ohle R, McIsaac SM, Woo MY, Perry JJ. Sonography of the Optic Nerve Sheath Diameter for Detection of Raised Intracranial Pressure Compared to Computed Tomography: A Systematic Review and Meta-analysis. J Ultrasound Med. 2015;34(7):1285-94. | US | Neurological | No | 47 |
| 73 | Wu L, Cao Y, Liao C, Huang J, Gao F. Diagnostic performance of USPIO-enhanced MRI for lymph-node metastases in different body regions: a meta-analysis. Eur J Radiol. 2011;80(2):582-9. | MRI | General interest | Yes | 47 |
| 74 | Sun Z, Davidson R, Lin CH. Multi-detector row CT angiography in the assessment of coronary in-stent restenosis: a systematic review. Eur J Radiol. 2009;69(3):489-95. | CTA | Cardiac | No | 46 |
| 75 | Pelgrim GJ, Dorrius M, Xie X, den Dekker MA, Schoepf UJ, Henzler T, et al. The dream of a one-stop-shop: Meta-analysis on myocardial perfusion CT. Eur J Radiol. 2015;84(12):2411-20. | CTA | Cardiac | Yes | 46 |
| 76 | Lu YY, Chen JH, Ding HJ, Chien CR, Lin WY, Kao CH. A systematic review and meta-analysis of pretherapeutic lymph node staging of colorectal cancer by 18F-FDG PET or PET/CT. Nucl Med Commun. 2012;33(11):1127-33. | PET, PET-CT | Abdominal/pelvic | No | 46 |
| 77 | Wu LM, Chen FY, Jiang XX, Gu HY, Yin Y, Xu JR. 18F-FDG PET, combined FDG-PET/CT and MRI for evaluation of bone marrow infiltration in staging of lymphoma: a systematic review and meta-analysis. Eur J Radiol. 2012;81(2):303-11. | PET, PET-CT, MRI | Musculoskeletal | Yes | 45 |
| 78 | Springer I, Dewey M. Comparison of multislice computed tomography with intravascular ultrasound for detection and characterization of coronary artery plaques: a systematic review. Eur J Radiol. 2009;71(2):275-82. | CTA | Cardiac | No | 45 |
| 79 | Wong KK, Fig LM, Gross MD, Dwamena BA. Parathyroid adenoma localization with 99mTc-sestamibi SPECT/CT: a meta-analysis. Nucl Med Commun. 2015;36(4):363-75. | SPECT-CT, CT | Head and neck | Yes | 45 |
| 80 | Hao R, Yuan L, Kan Y, Li C, Yang J. Diagnostic performance of 18F-FDG PET/CT in patients with fever of unknown origin: a meta-analysis. Nucl Med Commun. 2013;34(7):682-8. | PET-CT | General interest | No | 45 |
| 81 | Medina LS, Bernal B, Ruiz J. Role of functional MR in determining language dominance in epilepsy and nonepilepsy populations: a Bayesian analysis. Radiology. 2007;242(1):94-100. | fMRI | Neurological | No | 45 |
| 82 | Kianifar HR, Tehranian S, Shojaei P, Adinehpoor Z, Sadeghi R, Kakhki VR, et al. Accuracy of hepatobiliary scintigraphy for differentiation of neonatal hepatitis from biliary atresia: systematic review and meta-analysis of the literature. Pediatr Radiol. 2013;43(8):905-19. | Scintigraphy | Abdominal | No | 44 |
| 83 | Singer AD, Subhawong TK, Jose J, Tresley J, Clifford PD. Ischiofemoral impingement syndrome: a meta-analysis. Skeletal Radiol. 2015;44(6):831-7. | MRI | Musculoskeletal | No | 44 |
| 84 | Vilgrain V, Esvan M, Ronot M, Caumont-Prim A, Aubé C, Chatellier G. A meta-analysis of diffusion-weighted and gadoxetic acid-enhanced MR imaging for the detection of liver metastases. Eur Radiol. 2016;26(12):4595-615. | MRI | Abdominal | Yes | 44 |
| 85 | Sun Z, Ng KH. Diagnostic value of coronary CT angiography with prospective ECG-gating in the diagnosis of coronary artery disease: a systematic review and meta-analysis. Int J Cardiovasc Imaging. 2012;28(8):2109-19. | CTA | Cardiac | No | 44 |
| 86 | Chen YK, Yeh CL, Tsui CC, Liang JA, Chen JH, Kao CH. F-18 FDG PET for evaluation of bone marrow involvement in non-Hodgkin lymphoma: a meta-analysis. Clin Nucl Med. 2011;36(7):553-9. | PET, PET-CT | Musculoskeletal | No | 42 |
| 87 | Juneau D, Golfam M, Hazra S, Zuckier LS, Garas S, Redpath C, et al. Positron Emission Tomography and Single-Photon Emission Computed Tomography Imaging in the Diagnosis of Cardiac Implantable Electronic Device Infection: A Systematic Review and Meta-Analysis. Circ Cardiovasc Imaging. 2017;10(4). | PET-CT | Cardiac | No | 42 |
| 88 | Vorre MM, Abdulla J. Diagnostic accuracy and radiation dose of CT coronary angiography in atrial fibrillation: systematic review and meta-analysis. Radiology. 2013;267(2):376-86. | CTA | Cardiac | No | 42 |
| 89 | Cheng X, Li Y, Liu B, Xu Z, Bao L, Wang J. 18F-FDG PET/CT and PET for evaluation of pathological response to neoadjuvant chemotherapy in breast cancer: a meta-analysis. Acta Radiol. 2012;53(6):615-27. | PET, PET-CT | Breast | Yes | 41 |
| 90 | Chua AE, Ridley LJ. Diagnostic accuracy of CT angiography in acute gastrointestinal bleeding. J Med Imaging Radiat Oncol. 2008;52(4):333-8. | CTA | Abdominal/pelvic | No | 41 |
| 91 | Jie C, Rongbo L, Ping T. The value of diffusion-weighted imaging in the detection of prostate cancer: a meta-analysis. Eur Radiol. 2014;24(8):1929-41. | MRI | Pelvic | No | 40 |
| 92 | Barger RL, Jr., Nandalur KR. Diagnostic performance of dual-time 18F-FDG PET in the diagnosis of pulmonary nodules: a meta-analysis. Acad Radiol. 2012;19(2):153-8. | PET | Thoracic | No | 40 |
| 93 | Moore MM, Kulaylat AN, Hollenbeak CS, Engbrecht BW, Dillman JR, Methratta ST. Magnetic resonance imaging in pediatric appendicitis: a systematic review. Pediatr Radiol. 2016;46(6):928-39. | MRI | Abdominal | No | 39 |
| 94 | Meijer AB, O YL, Geleijns J, Kroft LJ. Meta-analysis of 40- and 64-MDCT angiography for assessing coronary artery stenosis. AJR Am J Roentgenol. 2008;191(6):1667-75. | CTA | Cardiac | No | 39 |
| 95 | Iared W, Shigueoka DC, Cristófoli JC, Andriolo R, Atallah AN, Ajzen SA, et al. Use of color Doppler ultrasonography for the prediction of malignancy in follicular thyroid neoplasms: systematic review and meta-analysis. J Ultrasound Med. 2010;29(3):419-25. | Doppler-US | Head and neck | No | 39 |
| 96 | Liu J, Xu Y, Wang J. Ultrasonography, computed tomography and magnetic resonance imaging for diagnosis of ovarian carcinoma. Eur J Radiol. 2007;62(3):328-34. | US, CT, MRI | Pelvic | Yes | 39 |
| 97 | Millet I, Taourel P, Ruyer A, Molinari N. Value of CT findings to predict surgical ischemia in small bowel obstruction: A systematic review and meta-analysis. Eur Radiol. 2015;25(6):1823-35. | CT | Abdominal | Yes | 39 |
| 98 | Foerster BR, Dwamena BA, Petrou M, Carlos RC, Callaghan BC, Pomper MG. Diagnostic accuracy using diffusion tensor imaging in the diagnosis of ALS: a meta-analysis. Acad Radiol. 2012;19(9):1075-86. | MRI | Neurological | No | 39 |
| 99 | Mir DI, Gupta A, Dunning A, Puchi L, Robinson CL, Epstein HA, et al. CT perfusion for detection of delayed cerebral ischemia in aneurysmal subarachnoid hemorrhage: a systematic review and meta-analysis. AJNR Am J Neuroradiol. 2014;35(5):866-71. | CTP | Neurological | No | 38 |
| 100 | Jens S, Koelemay MJ, Reekers JA, Bipat S. Diagnostic performance of computed tomography angiography and contrast-enhanced magnetic resonance angiography in patients with critical limb ischaemia and intermittent claudication: systematic review and meta-analysis. Eur Radiol. 2013;23(11):3104-14. | CTA, MRA | Vascular | Yes | 38 |

CT, computed tomography; CTA, CT angiography; echo, echocardiography; MR, magnetic resonance; MRA, MR angiography; MRI, MR imaging; fMRI, functional MRI; PET, positron emission tomography; SPECT, single-photon emission CT; US, ultrasound.
